# Hepatitis C virus infection is associated with high risk of breast cancer: a pooled analysis of 68,014 participants

**DOI:** 10.3389/fonc.2023.1274340

**Published:** 2023-10-13

**Authors:** Haiping Chen, Pei Du, Tianyao Yang, Xueyuan Xu, Tianyang Cui, Yuhang Dai

**Affiliations:** ^1^ Department of Infectious Diseases, Taizhou Hospital of Zhejiang Province Affiliated to Wenzhou Medical University, Taizhou, Zhejiang, China; ^2^ Department of Gynecology and Obstetrics, Guangzhou Panyu Central Hospital, Guangzhou, Guangdong, China; ^3^ Department of Thyroid and Breast Surgery, Tiantai People's Hospital, Taizhou, Zhejiang, China; ^4^ Department of Clinical Medical School, Taizhou University, Taizhou, Zhejiang, China; ^5^ Department of Gastroenterology, Taizhou Central Hospital (Taizhou University Hospital), Taizhou, Zhejiang, China

**Keywords:** breast cancer, hepatitis C virus, cumulative analysis, risk, prevalence

## Abstract

**Introduction:**

Breast cancer is the most common malignancy among women. Previous studies had shown that hepatitis C virus (HCV) infection might serve as a risk factor for breast cancer, while some studies failed to find such an association.

**Methods:**

In this study, we presented a first attempt to capture and clarify this clinical debate via a cumulative analysis (registration ID: CRD42023445888).

**Results:**

After systematically searching and excluding the irrelevant publications, five case-control or cohort studies were finally included. The synthetic effect from the eligible studies showed that patients with HCV infection had a significantly higher prevalence of breast cancer than non-HCV infected general population (combined HR= 1.382, 95%CI: 1.129 to 1.692, *P*=0.002). There was no evidence of statistical heterogeneity during this pooled analysis (*I^2 ^= *13.2%, *P*=0.33). The sensitivity analyses confirmed the above findings. No significant publication bias was observed among the included studies. The underlying pathophysiological mechanisms for this relationship might be associated with persistent infection/inflammation, host immune response, and the modulation of HCV-associated gene expression.

**Discussion:**

Though the causal association between HCV infection and breast cancer did not seem quite as strong, screening for HCV might enable the early detection of breast cancer and help to prevent the progression of the disease. Since the topic of this study remains a matter of clinical debate, further studies are still warranted to validate this potential association.

**Systematic review registration:**

https://www.crd.york.ac.uk/PROSPERO/, identifier CRD42023445888

## Introduction

According to the Cancer Statistics 2023 (1), breast cancer is still the most common malignancy among women, accounting for 31% of new diagnoses of female cancers. The estimated new cases of breast cancer are predicted at 297,790 which is more than two-fold of new diagnoses of lung and bronchus cancers (120,790 cases). Besides, breast cancer contributes the second greatest number of deaths in women, accounting for 15% of estimated deaths (43,170 cases) (1). There are projected to be more than 3 million new cases of breast cancer every year by 2040, as well as more than 1 million deaths per year from the disease (2). The frequency and the death of breast cancer are various in different races. It is reported that black women are 4% less likely to develop breast cancer than white women, but the mortality in black women is 40% higher than in white women (1). The tumorigenesis of breast cancer may be influenced by a variety of risk factors, these include, but are not limited to age, family history and hereditary factors, early menarche and late menopause, delayed or nulliparous fertility, long-term hormone replacement therapy, mammary gland hyperplasia and mammary duct ectasia, and environmental and lifestyle factors (3–6). With the progress of research, more and more risk factors have been identified for the development of breast cancer, such as the concomitant diseases, i.e., depression (7), meningioma (8), and endometriosis (9).

According to the current evidence, virus infection is significantly associated with the development of multiple malignancies, e.g. hepatitis virus and hepatocellular carcinoma (HCC), Epstein-Barr virus and nasopharyngeal carcinoma, human papillomavirus and cervical cancer, human T-lymphotropic virus 1 and T-cell lymphoma, and human cytomegalovirus (HCMV) and glioblastoma (10, 11). Interestingly, both Epstein-Barr virus and human papillomavirus infections are the risk factor for breast cancer (12, 13). Type C viral hepatitis is one of the common viral-mediated infectious diseases deriving from the liver. Mounting studies have implied that chronic hepatitis C virus (HCV) infection may cause the tumorigenesis of HCC as well as the extrahepatic malignancies (i.e., gastrointestinal cancers, lymphoma, lung cancer, urologic malignancies, and gynecologic cancers) on the account of the persistent inflammation induced by HCV infection (14–19).

In recent years, there has been an increasing amount of attention paid to the potential association between HCV and the risk of breast cancer (20). A nationwide cohort developed by CHENG et al. (21) demonstrated that untreated HCV infection (hazard ratio [HR]= 1.701: 95% CI: 1.205-2.4) was associated with the incidence of breast cancer. The authors further observed that a higher risk of breast cancer was detected in those patients who were <49 years (HR= 2.193: 95% CI: 1.097-4.384) (21). However, several related studies did not support such a positive relationship between HCV and breast cancer. Swart A et al. (22) showed that the number of breast cancer in the HCV-positive cohort was comparable to that of the control group. In line with Swart A’s findings, a few relevant studies also failed to find a significant association between HCV infection and the high risk of breast cancer (23, 24). These studies were designed to investigate the prevalence of breast cancer in patients with HCV. On the contrary, Liu et al. (25) conducted a study that investigated the prevalence of HCV in breast cancer patients. The results showed that the prevalence of HCV in patients with breast cancer was not significantly higher than that of the cancer-free inpatients (25).

Based on the above evidence, the association between chronic HCV infection and the risk of breast cancer is still controversial. Therefore, a systematic review and meta-analysis of the current evidence is urgently needed to evaluate this potential link between HCV-infected persons and the development of breast cancer. In this study, we presented a first attempt to capture and clarify this unrevealed clinical issue via a cumulative analysis.

## Methods

The PRISMA (Preferred Reporting Items for Systematic Reviews and Meta-Analyses) guidelines were followed for the present systematic review and cumulative analysis. **Supplementary Table 1** listed the PRISMA checklist. In addition, this study also registered with the PROSPERO (ID: CRD42023445888). More details of the methodology of this cumulative study could be found in PROSPERO. The following literature search, study selection, inclusion/exclusion criteria, and data extraction were conducted by two authors independently. Any ambiguities could be resolved by a third author.

### Data Sources and search strategy

Four commonly used electronic databases, i.e., MEDLINE (PubMed), the EMBASE, Cochrane Library, and PsychINFO databases, were systematically retrieved to identify the qualified studies. Those potential studies covered the period between the inception of the four databases and May 1, 2023. This review only included English-language studies. Based on searches in the MEDLINE database, the following terms were used in combinations: ((((((((((((((((((((((((((((((((((((((“Breast Neoplasms”[Mesh]) OR (Breast Neoplasm)) OR (Neoplasm, Breast)) OR (Breast Tumors)) OR (Breast Tumor)) OR (Tumor, Breast)) OR (Tumors, Breast)) OR (Neoplasms, Breast)) OR (Breast Cancer)) OR (Cancer, Breast)) OR (Mammary Cancer)) OR (Cancer, Mammary)) OR (Cancers, Mammary)) OR (Mammary Cancers)) OR (Malignant Neoplasm of Breast)) OR (Breast Malignant Neoplasm)) OR (Breast Malignant Neoplasms)) OR (Malignant Tumor of Breast)) OR (Breast Malignant Tumor)) OR (Breast Malignant Tumors)) OR (Cancer of Breast)) OR (Cancer of the Breast)) OR (Mammary Carcinoma, Human)) OR (Carcinoma, Human Mammary)) OR (Carcinomas, Human Mammary)) OR (Human Mammary Carcinomas)) OR (Mammary Carcinomas, Human)) OR (Human Mammary Carcinoma)) OR (Mammary Neoplasms, Human)) OR (Human Mammary Neoplasm)) OR (Human Mammary Neoplasms)) OR (Neoplasm, Human Mammary)) OR (Neoplasms, Human Mammary)) OR (Mammary Neoplasm, Human)) OR (Breast Carcinoma)) OR (Breast Carcinomas)) OR (Carcinoma, Breast)) OR (Carcinomas, Breast)) AND (((((“Hepacivirus”[Mesh]) OR (Hepatitis C virus)) OR (Hepatitis C viruses)) OR (HCV)) OR (Hepatitis C)). A manual search of the reference lists was also conducted to identify further eligible studies. The features of the included studies were displayed in **Table 1**, presenting the characteristics of the included studies.

**Table 1:** Characteristics of the five included studies.

Study	Study area	Study design	Mean age (years)	Study group case/total	Control group case/total	HR with 95%CI	Variable adjustment
Larrey (13) 2010	France	Case– control	21-84	17/294	5/107	1.24 (0.47-3.27)	NA
Su (12) 2011	Chinese Taipei	Cohort	A broad age	56/234	1760/8862	1.21 (0.96-1.52)	Age, residential area, occupation, urbanization, and income
Hwang (14) 2014	USA	Cohort	51.5 ± 15.95	3/35	105/2295	1.87 (0.62-5.62)	HIV, injection drug use, hemodialysis, hemophilia, and other liver conditions
CHENG-1 (15) 2022	Chinese Taipei	Cohort	A broad age	NA/14584	NA/14584	1.701 (1.205-2.4)	Liver cirrhosis, COPD, ESRD, DM, hypertension, dyslipidemia, cardiovascular events, and stroke
CHENG-2 (15) 2022	Chinese Taipei	Cohort	< 49 years	NA/18230	NA/14584	2.193 (1.097-4.384)	Liver cirrhosis, COPD, ESRD, DM, hypertension, dyslipidemia, cardiovascular events, and stroke
Loosen (15) 2022	Germany	Cohort	48.4 ± 19.2	NA/7667	NA/15706	1.04 (0.6-1.81)	Age, diabetes, obesity

S, Study group: patients with HBV or HCV infection; C, Control group; the healthy general population without HBV/HCV infection; NA, Not available; GC, Gastric cancer; HR, Hazard ratio; CI, Confidence interval; COPD, Chronic obstructive pulmonary disease; ESRD, End-stage renal disease; DM, Diabetes mellitus.

### Assessments of HCV and breast cancer

HCV infection and breast cancer were confirmed and classified by using the International Classification of Diseases (ICD) codes and standards of the World Health Organization (WHO). The diagnosis of HCV infection was validated based on the presence of anti-HCV seropositivity for at least 6 months or liver histology. The diagnosis of breast cancer was confirmed by histopathology, clinical expression, and mammography.

### Inclusion criteria

Any studies reporting the association between HCV infection and breast cancer were considered to be eligible, reporting either the prevalence of breast cancer in HCV patients or the prevalence of HCV in breast cancer patients. In addition, those studies providing a hazard ratio (HR), odds ratios (OR), or relative risk (RR) with the 95% confidence intervals (CI) that reported the relationship between HCV and breast cancer were also considered to be eligible. The scientific question for guiding this study was: Is there a positive association between HCV infection and breast cancer? The inclusion criteria for this study followed the PICOS standard: Patient (HCV infection patients with breast cancer or breast cancer with HCV), Intervention (diagnosis of breast cancer or HCV), Comparison (compared with the control subjects: either healthy population without HCV infection or those diagnosed with benign breast diseases), Outcome (the prevalence of breast cancer or HCV infection), and Study design (any study designs).

### Exclusion criteria

The exclusion criteria used in this study were: (a) the types of study belonged to review, comments, or case reports; (b) duplicated data derived from the same samples or the same scientific question; (c) non-human experimental studies; (d) since the present study is designed for evaluating whether HCV is an independent risk factor for breast cancer, thus those study samples presented with co-infected with both HBV and HCV being removed.

### Data extraction

To extract the essential data from each included study, we designed a data collection form. The following items were extracted, including the first authors’ names, publication year, country/region, study design, mean age of the participants, the number of breast cancer cases in the HCV group and the non-HCV group or HCV cases in the breast cancer group and the non-cancer group, HR with its 95%CI, and the variable adjustments.

### Quality assessment

Newcastle-Ottawa Scale (NOS) was applied to assess the methodological quality of cohort studies or case-control studies. The NOS checklist includes nine items, in which gains scores of 0–3, 4–6, and 7–9 represent low quality, moderate quality, and high quality, respectively.

### Statistical methodology

In order to conduct this cumulative analysis, STATA version 13.0 for Windows (Stata Corp LP, College Station, USA) was used. A quantitative assessment of the strength of the association between HCV infection and breast cancer was conducted by combining the overall HRs with 95% CIs for all the included studies. A two-tailed *P* value of 0.05 was assumed to indicate statistical significance. Statistical tests for heterogeneity were conducted using *I* ^2^ statistics and Cochrane *Q* statistics. Heterogeneity was considered substantial (statistical significance) when *I* ^2^ > 50% or the *P*-value of the *Q* test < 0.10. Rather than a fixed-effects model, a random-effects model was applied in this study due to a high probability of variability in study design and demographic characteristics. To further identify the potential sources of heterogeneity between studies, sensitivity analyses were conducted. For an evaluation of publication bias, the funnel plot, Begg’s rank-correlation test, and Egger’s regression asymmetry test were conducted.

## Results

### Literature search

**Figure 1:**
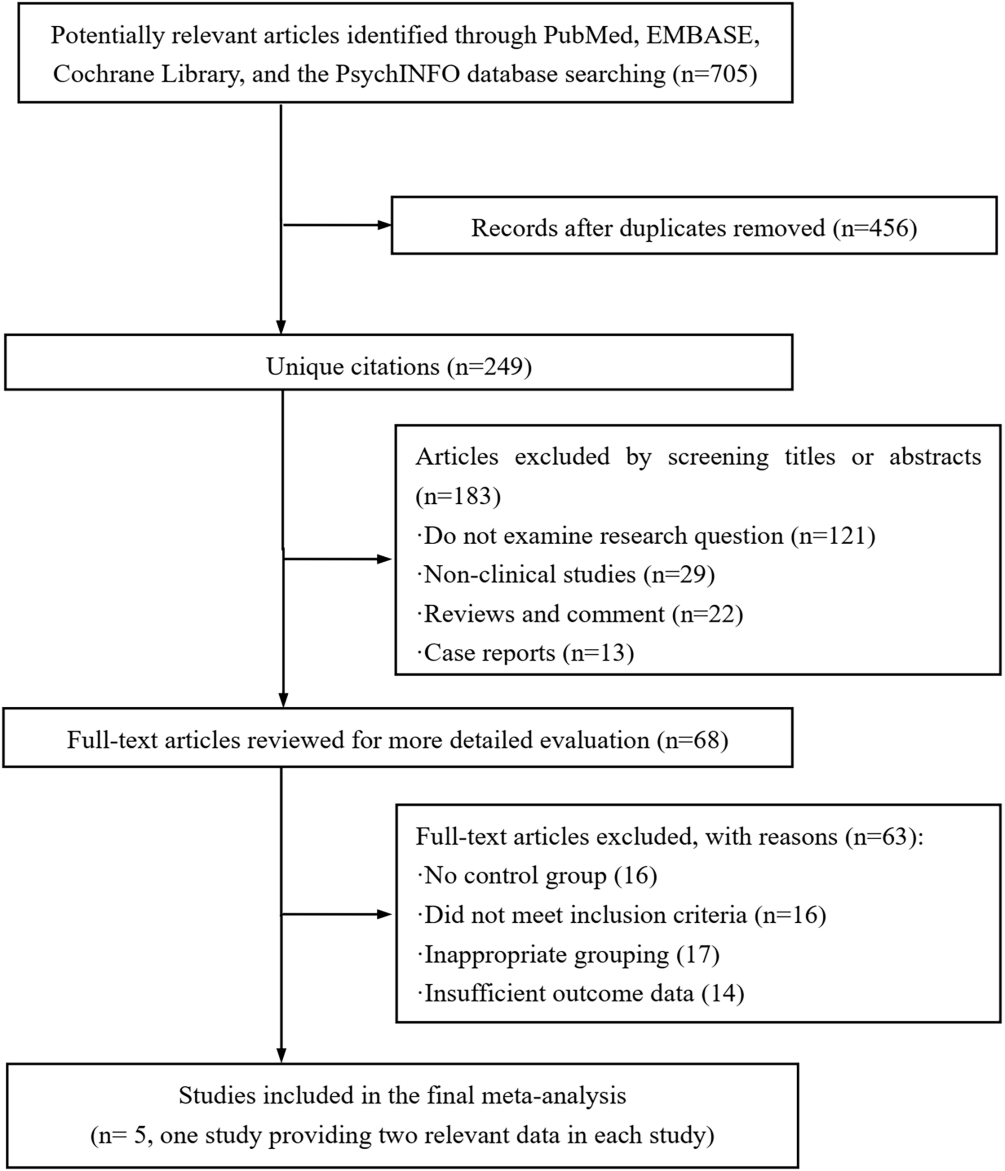
Flow chart of study selection.

A flow chart of the selection process for identifying the eligible articles could be found in **Figure 1**. During the initial search of the four databases, 705 articles were detected. After validating the duplicates and those studies did not examine the targeted research question, non-clinical studies, review articles, comments, and case reports, 637 publications were removed and the remaining 68 potential articles were retrieved for the full-text review. Among the remaining studies, 63 publications were eliminated due to lacking a control group, failure to meet the inclusion criteria, inappropriate grouping, and insufficient outcome data. Finally, five studies (21, 26–29) were included in this cumulative analysis. Of note, CHENG et al.’s study (21) provided additional data related to the young age of the patients, which was set as CHENG-1 and CHENG-2.

### Study characteristic

The publication date of the eight included studies ranged from 2010 to 2022. The age of the participants ranged from 21 to 84 years. In the aspect of geographical area, three, three, and two studies were conducted in Asia, Europe, and Africa, respectively. The study design of the eight included studies was either cohort or case-control. The sample size ranged from 158 to 32,814, with a total of 68,014 participants. The variable adjustments in the included studies included age, residential area, occupation, urbanization, income, human immunodeficiency virus (HIV) infection, injection drug use, hemodialysis, hemophilia, chronic obstructive pulmonary disease (COPD), end-stage renal disease (ESRD), diabetes mellitus (DM), hypertension, dyslipidemia, cardiovascular events, stroke, and obesity. The characteristics and the HR with 95%CI of the eight included studies were summarized in **Table 1**.

### Study quality

According to the scoring criteria of the NOS, three of the included studies were judged to be of high quality and the remaining two included studies were of moderate quality. In all, 60% (3/5) of the included studies were considered to have high methodological quality. **Supplementary Table 2** provided a detailed scoring of the study quality.

### Cumulative analysis

**Figure 2:**
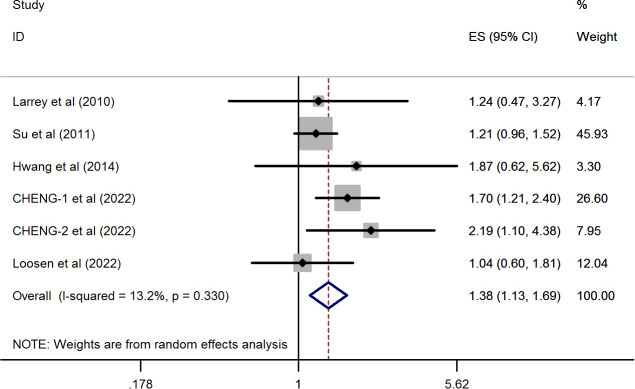
Forest plots of the pooled analysis of the included studies reporting the prevalence of breast cancer in patients with chronic HCV infection.

As shown in **Figure 2**, the synthetic effect from five included studies showed that a significantly higher prevalence of breast cancer was observed in patients with HCV infection than those with negative anti-HCV tests (pooled HR = 1.382, 95%CI: 1.129 to 1.692, *P*=0.002) by conducting a random-effects model. There was no evidence of statistical heterogeneity during this combined analysis (*I^2 ^= *13.2%, *P*=0.33). These results suggested that the association between HCV infection and the risk of breast cancer was explicit.

### Sensitivity analysis

**Figure 3:**
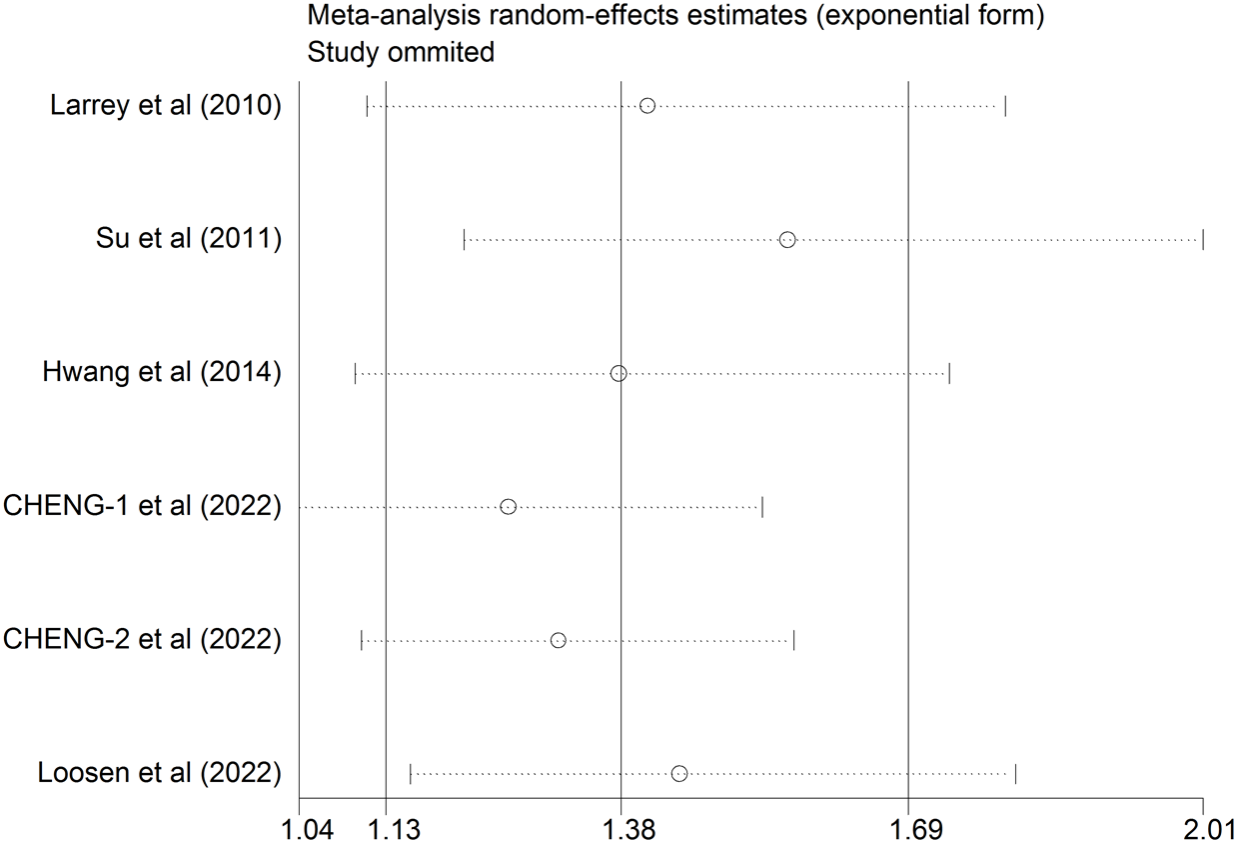
Sensitivity analysis after each study was excluded by turns.

In order to determine how an individual study influenced a newly calculated overall HR, a sensitivity analysis was conducted. As shown in **Table 2** and **Figure 3**, the positive association between HCV infection and risk of breast cancer was consistent after removing any one of the included studies. The new HR ranged from 1.207 (95%CI: 0.96 to 1.455, *P*<0.001) to 1.422 (95%CI: 1.032 to 1.812, *P*<0.001). Besides, there was no substantial change in the heterogeneity test after eliminating anyone from the study (*I* ^2^ ranged from 0.0% to 4.4%, all *P >*0.1). Based on these results, it appeared that no single study dominated the pooled HR and heterogeneity among studies.

**Table 2:** Sensitivity analysis in the five included studies reporting HCV and risk of breast cancer.

Study omitted	RR (95% CI) for remainders	Heterogeneity	
		*I* ^2^	*P*
Larrey et al. (2010)	1.291 (1.039, 1.542) *P*<0.001	4.4%	0.382
Su et al. (2011)	1.422 (1.032, 1.812) *P*<0.001	0.0%	0.492
Hwang et al. (2014)	1.275 (1.045, 1.504) *P*<0.001	0.0%	0.41
CHENG-1 et al. (2022)	1.207 (0.96, 1.455) *P*<0.001	0.0%	0.745
CHENG-2 et al. (2022)	1.262 (1.031, 1.493) *P*<0.001	0.0%	0.561
Loosen et al. (2022)	1.32 (1.073, 1.567) *P*<0.001	0.0%	0.48

HR, hazard ratio; CI, confidence interval.

### Publication bias

**Figure 4:**
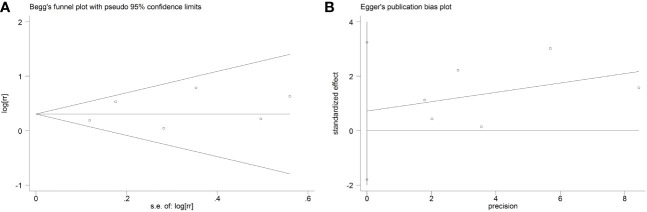
Publication bias analyses. **(A)** Begg’s test; **(B)** Egger’s test.

As shown in **Figure 4**, both the Begg’s and Egger’s tests demonstrated that there was no significant publication bias was observed among the included studies (Begg’s, *P* > |z| = 0.707; Egger, *P* > |t| = 0.471, 95%CI: -1.801 to 3.247).

## Discussion

According to the available published data, several studies have assessed the association between HCV infection and the development of breast cancer. However, the relevant studies presented with the inconsistent results on this relationship. In this study, we firstly to clarify this conspicuous issue by quantifying the HR from each related study through a meta-analysis. Based on the combined HR from the five included studies reporting the prevalence of breast cancer in HCV-infected patients, the results revealed that that anti-HCV positive patients were at 1.38-fold higher risk of the development of breast cancer than the healthy population without HCV infection with a statistical significance (synthetic HR= 1.38, 95%CI: 1.129 to 1.692, *P*=0.002). No substantial heterogeneity was identified in this pooled analysis. Subsequent sensitivity analysis and variable adjustments also confirmed this finding. Based on the above evidence, a positive association between HCV infection and breast cancer development was detected.

Since there was a positive association between HCV infection and the risk of breast cancer, the potential pathophysiological mechanisms of HCV-induced breast cancer should be noted. For example, anti-HCV positivity was found to be correlated to the development of secondary breast cancer (30). Hussein et al. reported that the prevalence of HCV seropositivity was 6-fold greater in women with breast cancer (<45 years) than in adults of the same age without breast cancer diagnoses (31). Similar to Hussein et al.’s findings, a previous case-control study (27) also suggested that HCV-positive women who age <50 years had a 2-fold greater risk of developing breast cancer than the HCV-negative women with comparable age (OR = 2.03, 95%CI = 1.23 to 3.34). Therefore, it is imperative to uncover the pathomechanisms of the HCV-mediated breast cancer. According to the current evidence, the underlying mechanisms that existed in this potential relationship might be associated with multiple etiologies, including persistent infection/inflammation, host immune response, and the modulation of HCV-associated gene expression (27, 30).

The HCV infection promoted and maintained chronic inflammation in the infected sites, mainly in the liver but also in some organs and tissues other than the liver (32). This is due to viral antigens and genomes that have been detected in extra-hepatic tissues (33). Persistent inflammation induced by HCV infection causes the cancerous transformation of the extra-hepatic organs, which may be correlated to the response to a progressive reorganization of their structure (33). On the other hand, chronic inflammation may cause the genetic instability and arise genetic and epigenetic alterations in cells, resulting in carcinogenesis. It was reported that HCV infection could cause lymphoproliferation by inducing cytokine production (34), while aberrant lymphoproliferation had the potential to transfer as the tumor infiltrating lymphocytes (TILs) (35). TILs are the recognized oncogenic factors for the development and progression of breast cancer (36). Therefore, HCV-mediated inflammatory cytokines may induce an indirect carcinogen for breast cancer.

It was suggested that HCV could maintain persistent infection through immune evasion mechanisms (37). Thus, systemic impairment of immune function induced by HCV might also play role in the tumorigenesis of breast cancer. As reported, HCV-associated antigens, genome or replicative sequences were detected in T- and B-lymphocytes (38), indicating HCV might involve in the host immune response. Chronic HCV infection may induce immunocompromised status on account of the neutrophil or T-cell dysfunction (39). As a result of chronic antigenic stimulation by HCV, B lymphocytes expand clonally, producing monoclonal and polyclonal antibodies and producing immune complexes (40). Mounting evidence suggests that HCV not only plays an oncogenetic role in cancer development but is also involves in immunity and autoimmunity disorders (41). There are pathological, biochemical, and immunological abnormalities associated with HCV, which indicate its potential tumorigenicity (42).

Modulation of HCV-associated gene expression might also play role in HCV-mediated breast cancer. Attallah et al. (43) suggested that HCV infection in breast cancer patients was correlated to high levels of serum fibronectin and circulating HCV-NS4 expressions. HCV was also found to inactivate the cancer suppressor proteins, such as retinoblastoma proteins (Rb) and p53, affecting cell cycle, cell viability, and genome stability (44). HCV nonstructural proteins causing breast cancer progression might be associated with the downregulation of Rb (43). Moreover, HCV nonstructural proteins could form a complex with Rb, resulting in the reduction of Rb and ultimately induced cancer cell proliferation (45). It was reported that HCV could encode several viral proteins, i.e., Core- and NS5A, thus interacting with intracellular cascades pathways and functioning in the oncogenesis of breast cancer (33). In addition to the above potential pathophysiological mechanisms, metabolic alterations subsequent to HCV infection, might also play roles in the induction of breast cancer (21, 46). As reported, several HCV-mediated metabolic events could not be reversed, even after viral clearance (46). The metabolic factors might involve in the development of HCV-associated breast cancer.

For the first time, we tried to clarify the controversial clinical findings with the topic: “Is HCV infection a risk factor for breast cancer?”. We found that patients with HCV infection had a significantly higher prevalence of breast cancer than non-HCV healthy controls. The carcinogenic effects on breast cancer development induced by HCV infection might be associated with the persistent infection/inflammation, immune escape, and the modulation of HCV-associated gene expression. However, HCV might be not a strong promoter of breast cancer due to only 1.38-fold higher risk was detected. The causal association between HCV infection and breast cancer remains further investigation due to the results were derived from limited included studies. Besides, the study sample and study design varied across the included studies, which might interfere the exact association between HCV infection and breast cancer. Since a positive association between HCV infection and risk of breast cancer is detected, patients with or without treatment for HCV might affect the risky of breast cancer development. Among the five included studies, only one study (Larrey et al.) (26) reported the relationship between past or ongoing treatment of HCV (70%) or never treated HCV (30%) and the risk of breast cancer. However, Larrey et al.’s study did not show the independent prevalence of breast cancer in patients with past/ongoing treatment of HCV or never treated HCV. Therefore, we could not judge what was the difference on the strength of the association between HCV infection and risk of breast cancer in the two groups. Chronic HCV is currently treatable with several direct-acting antivirals (DAAs) that can target various HCV genotypes, stages of liver disease, and comorbidities (47). At present, DAAs and interferon-free and ribavirin-free regimens are used for the treatment of HCV infection. Since HCV infection may increase the risk of breast cancer, it is speculated that patients with HCV antiviral therapies may have a low risk of breast cancer than those without HCV treatment. This hypothesis was evidenced by several studies demonstrated that antiviral therapies for HCV might improve the outcomes of HCV-associated extrahepatic diseases, such as cardiovascular risk profile (48) and renal function (49). Of note, however, a case series study (50) demonstrated that a possible relationship between treatment with DAAs and development of extrahepatic malignancies, including breast cancer. Therefore, further studies are needed to explore whether the specific treatments for HCV will increase or reduce the risk of breast cancer.

## Conclusion

In summary, the present cumulative study demonstrated that patients with HCV infection were at a 1.38-fold higher risk of the development of breast cancer than the healthy population with a statistical significance. However, it should be acknowledged that the direction of causality between HCV infection and risk of breast cancer was not so clear due to limited studies were included and all of them had a retrospective design. Therefore, future prospective, well-designed cohorts with large samples and strict inclusion criteria are still warranted to better validate the relationship between HCV infection and breast cancer.

## Data availability statement

The original contributions presented in the study are included in the article/**Supplementary material**, further inquiries can be directed to the corresponding author/s.

## Author contributions

HC: Data curation, Investigation, Writing – original draft. PD: Formal Analysis, Writing – review & editing. XX: Data curation, Writing – original draft. TC: Methodology, Writing – review & editing. YD: Writing – original draft, Writing – review & editing. TY: Supervision, Writing – review & editing.

## Supplementary material

Supplementary Table 1 PRISMA Checklist of 2020.
