# Supplementary material for: Hepatitis C virus infection is associated with high risk of breast cancer: a pooled analysis of 68,014 participants
Source: Front Oncol. 2023 Oct 13;13:1274340. doi: 10.3389/fonc.2023.1274340 (PMC10613072; doi:10.3389/fonc.2023.1274340)
Supplement: Supplementary file 2 [file Table_2.doc]

| Study | Selection | | | | Comparability | | Exposure/Outcome | | | Total scores |
| --- | --- | --- | --- | --- | --- | --- | --- | --- | --- | --- |
|  | 1 | 2 | 3 | 4 | 5 | 6 | 7 | 8 | 9 |  |
| Larrey et al (2010) | Yes | Yes | Yes | Yes | No | Yes | No | Yes | No | 6 |
| Su et al (2011) | Yes | Yes | Yes | Yes | Yes | Yes | No | Yes | No | 7 |
| Hwang et al (2014) | Yes | Yes | Yes | Yes | Yes | Yes | Yes | No | No | 7 |
| CHENG et al (2022) | Yes | Yes | Yes | Yes | Yes | Yes | Yes | No | No | 7 |
| Loosen et al (2022) | Yes | Yes | Yes | Yes | Yes | No | Yes | No | No | 6 |

Supplementary Table 2. Newcastle-Ottawa Scale assessment of the quality of the case-control and cohort studies.

NOTE: 1. indicates that the exposed cohort was representative of the population; 2. Indicates that the non-exposed cohort was drawn from the same population; 3. Indicates that the exposure ascertainment was from secure records or a structured interview; 4. Indicates that outcome of interest was not present at start of study; 5. Indicates that the cohorts were comparable for age and sex; 6. Indicates that the cohorts were comparable on all additional factor(s) reported; 7. Indicates that outcome was assessed from a secure record; 8. Indicates that follow-up was long enough for outcomes to occur; 9. Indicates that follow-up was complete.
